# Targeting Adenosine Receptor by Polydeoxyribonucleotide: An Effective Therapeutic Strategy to Induce White-to-Brown Adipose Differentiation and to Curb Obesity

**DOI:** 10.3390/ph14080728

**Published:** 2021-07-27

**Authors:** Federica Mannino, Giovanni Pallio, Alessandra Bitto, Domenica Altavilla, Letteria Minutoli, Violetta Squadrito, Vincenzo Arcoraci, Domenico Antonio Giorgi, Igor Pirrotta, Francesco Squadrito, Natasha Irrera

**Affiliations:** 1 Department of Clinical and Experimental Medicine, University of Messina, Via C. Valeria, 98125 Messina, Italy; fmannino@unime.it (F.M.); gpallio@unime.it (G.P.); abitto@unime.it (A.B.); lminutoli@unime.it (L.M.); varcoraci@unime.it (V.A.); domenicoantonio.giorgi@unime.it (D.A.G.); igor.pirrotta@unime.it (I.P.); nirrera@unime.it (N.I.); 2 Department of Biomedical, Dental, Morphological and Functional Imaging Sciences, University of Messina, Via C. Valeria, 98125 Messina, Italy; daltavilla@unime.it; 3 Department of Human Pathology and Evolutive Age “Gaetano Barresi”, University of Messina, Via C. Valeria, 98125 Messina, Italy; violettasquadrito@gmail.com

**Keywords:** A_2A_ receptor, polydeoxyribonucleotide, adipocytes, browning process, *Ucp1*

## Abstract

Obesity is a worldwide chronic metabolic disease characterized by an abnormal fat accumulation and represents one of the main risk factors for several diseases. White adipose tissue is the primary site for energy storage in the form of triglycerides, whereas brown adipose tissue does not store energy-providing lipids but rather dissipates it by producing heat. White-to-brown adipocyte trans-differentiation could represent a new target of anti-obesity strategies and result in fat reduction. Previous studies indicated that adenosine receptor activation induces trans-differentiation of white adipocytes to brown adipocytes. The aim of this study was to evaluate the effects of polydeoxyribonucleotide (PDRN), an A_2Ar_ receptor agonist, in an in vitro model of browning. Mouse 3T3-L1 pre-adipocytes were differentiated in mature adipocytes with specific culture media and then treated with PDRN (10 µg/mL), PDRN + ZM241385 (1 µM), CGS21680 (1 µM) and CGS + ZM241385 for 24 h. Cell viability was studied by MTT assay, and browning induction was evaluated by Oil Red O staining and by RT-qPCR to study gene expression of browning markers. PDRN, as well as CGS21680, reduced the accumulation of lipids, cell volume and lipid droplet size; increased the expression of UCP1, PRDM16 and DIO2, considered as browning markers; and reduced the expression of FASn and FABP4, considered as whitening markers. In addition, PDRN decreased leptin expression and enhanced adiponectin mRNA levels. All these effects were abrogated when PDRN was co-incubated with the A_2Ar_ antagonist ZM241385. In conclusion, these results suggest that PDRN is able to induce the white-to-brown adipose differentiation through A_2Ar_ stimulation. Since PDRN is a safe drug already available in the market for other therapeutic indications, its “anti-obesity” potential warrants investigation in a clinical scenario.

## 1. Introduction

The significant imbalance of calorie intake and energy expenditure is responsible for obesity, which is a worldwide health problem characterized by an excessive amount of body fat [1]. This altered equilibrium associated with fat accumulation and the increased release of pro-inflammatory cytokines and adipokines by adipocytes make obesity an important risk factor for multiple metabolic and systemic complications, including type 2 diabetes, dyslipidemia, non-alcoholic fatty liver disease, cardiovascular diseases and even some cancers [2]. In fact, obesity represents a growing problem both for the affected patients and health care systems. In this context, obesity management is essential, but current therapeutic strategies mainly attempt to avoid the appearance of obesity-associated diseases [3]. Currently, obesity research is also based on the possibility of converting white adipose tissue (WAT) to brown adipose tissue (BAT), thus promoting the so-called “browning” process that might fight obesity and its related diseases [4].

BAT plays a pivotal role in metabolism, energy expenditure (EE) and adaptive thermogenesis. In contrast with white adipocytes, which store energy-providing lipids, brown adipocytes do not store energy but, on the contrary, produce heat by energy dissipation [5].

BAT is innervated by noradrenergic parenchymal fibers through gap junctions, which are responsible for the functional activation of brown adipocytes [6]. Noradrenaline acts on BAT through β3-adrenoceptors, thus inducing lipolysis, mitochondriogenesis and uncoupling protein 1 (UCP1) synthesis [7]. UCP1 uncouples oxidative phosphorylation from ATP synthesis and dissipates the energy provided by triglycerides in the form of heat [8]. As WAT has a “reserve” function, and even though WAT and BAT together form the adipose tissue, they differently express UCP1, in favor of BAT [9]. The adipose organ undergoes whitening and browning in obese animals. These processes are accompanied by UCP1 decrease/increase, causing whitening and browning, respectively [10].

Adenosine is an essential nucleoside involved in energy production and regulates different processes in several tissues, including adipose tissue. The biological effects of adenosine are the result of the interaction with adenosine receptors (A1, A2A, A2B and A3) which are G protein-coupled receptors [11]. These receptors have different affinities for adenosine: in particular, A1R and A2AR have high affinity for adenosine, while A2BR and A3R have relatively lower affinity [12]. Adenosine receptors are widely distributed in the body, and different scientific results have demonstrated that adipocytes also express adenosine receptors [13]. In particular, A2A receptor expression was detected in both BAT and WAT, but its modulation seems to be essential for BAT regulation, as A2A receptor activation may induce trans-differentiation of white adipocytes to brown adipocytes in WAT [13,14]. Therefore, targeting A2A receptors might be considered a potential therapeutic approach for stimulating the browning process. 

Polydeoxyribonucleotide (PDRN) is a natural compound composed of a mixture of deoxyribonucleotides polymers whose mechanism of action is the consequence of A2A receptor stimulation [15]. Previous experiments have demonstrated PDRN efficacy in treating inflammation and promoting tissue remodeling [16,17]. It is a safe drug, already available in the market for other therapeutic indications. The present study aimed to evaluate whether PDRN might also stimulate the browning process through adenosine receptor stimulation in 3T3-L1 adipocytes cell line.

## 2. Results

### 2.1. PDRN Does Not Affect Cell Viability 

**Figure 1:**
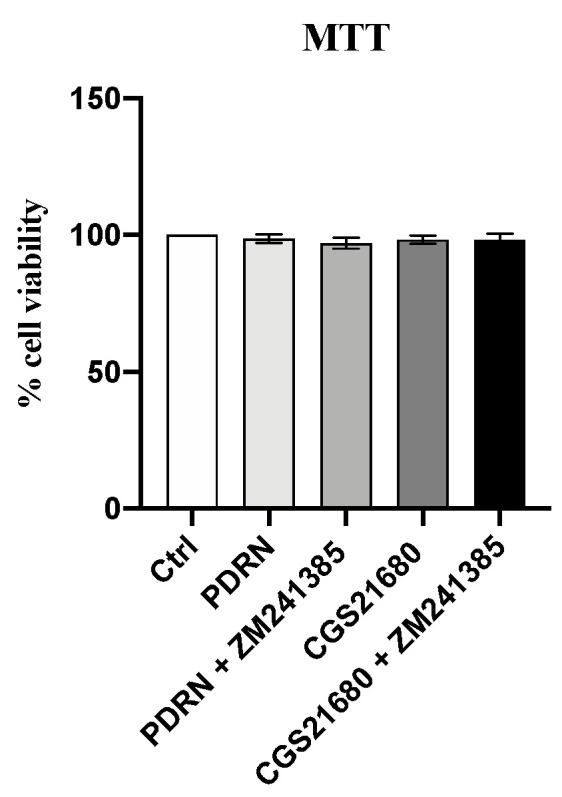
The graph shows cytotoxicity in 3T3-L1 cells by MTT assay following 24 h of treatment with PDRN, PDRN + ZM241385, CGS21680 and CGS21680 + ZM241385. Values are expressed as the means and SD; *n* = 3 experiments.

PDRN cytotoxicity was evaluated on the 3T3-L1 cell line by MTT assay. One hundred percent of viability was observed in control cells following 24 h. PDRN treatment alone or with ZM 241385 did not affect cell viability, thus demonstrating that polynucleotides did not have a cytotoxic effect. As expected, also CGS21680 alone or with ZM 241385 did not show any toxicity (Figure 1).

### 2.2. PDRN Promotes Trans-Differentiation from White Adipocytes to Beige Adipocytes

**Figure 2:**
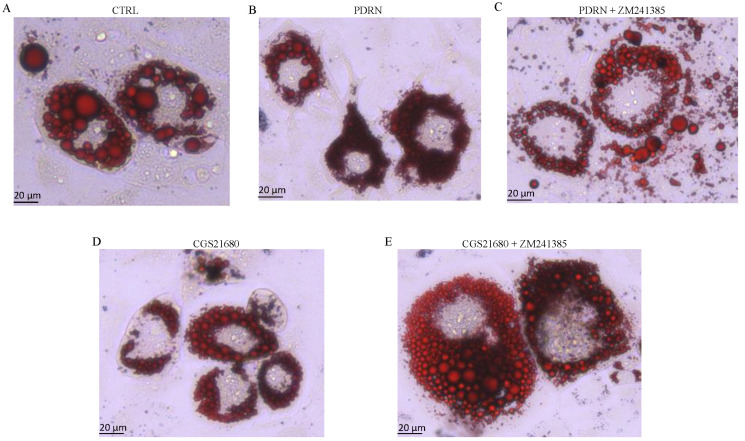
The panel shows differentiated adipocytes stained by Oil Red O staining of CTRL (**A**), PDRN (**B**), PDRN + ZM241385 (**C**), CGS21680 (**D**) and CGS21680 + ZM241385 (**E**). Scale bar 20 µm.

**Figure 3:**
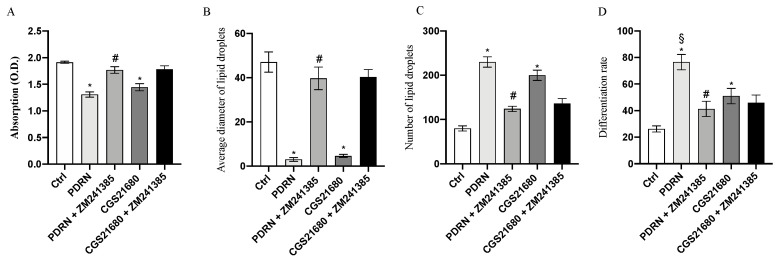
The graphs represent Od 500 Oil Red O values (**A**), lipid droplet size (**B**), number of lipid droplets (**C**) and adipocyte differentiation rate (**D**) obtained from each group. Values are expressed as the means and SD. *N* = 3 experiments; * *p* < 0.0001 vs. CTRL; # *p* < 0.0001 vs. PDRN; *§ p* < 0.001 vs. CGS21680.

Lipid accumulation in 3T3-L1 cell line was qualitatively evaluated by Oil Red O staining. After 14 days of differentiation, mature adipocytes were treated with 10 µg/mL of PDRN for 24 h. Based on microscopic observation, the Oil Red O staining demonstrated a significant reduction in both cell volume and lipid droplet size compared to untreated cells. Moreover, since the diameter of lipid droplets was significantly reduced, their number was increased following PDRN treatment together with the differentiation rate, as shown in Figure 2 and Figure 3. The same results were observed following CGS21680 treatment, thus confirming A_2A_ receptor involvement in the trans-differentiation process (Figure 2 and Figure 3). Moreover, PDRN and CGS21680 treatment caused a significant reduction in lipid accumulation as demonstrated by OD500 Oil Red O value calculation (Figure 3). Co-incubation with ZM 241385 abrogated the positive effect of both CGS and PDRN, confirming that PDRN acts through adenosine receptor modulation (Figure 2 and Figure 3).

### 2.3. Effect of PDRN on Oxygen Consumption 

**Figure 4:**
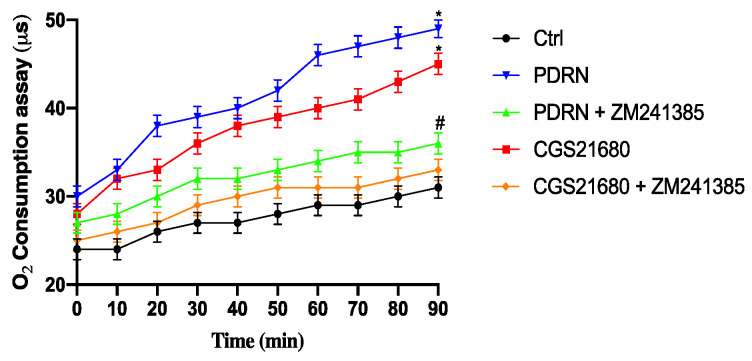
The graph represents the oxygen consumption in the 3T3L1 cell line. Values are expressed as the means and SD. *N* = 3 experiments; * *p* < 0.0001 vs. CTRL; # *p* < 0.0001 vs. PDRN.

Oxygen consumption was significantly increased in cells treated with PDRN or CGS21680 compared to controls. ZM241385 co-incubation with PDRN and CGS21680 abrogated the enhancement of oxygen consumption mediated by the treatment with both adenosine receptor agonists, confirming that PDRN was able to increase metabolic activity, as observed in brown adipocytes, through adenosine receptor modulation (Figure 4).

### 2.4. PDRN Promotes the Browning Process

**Figure 5:**
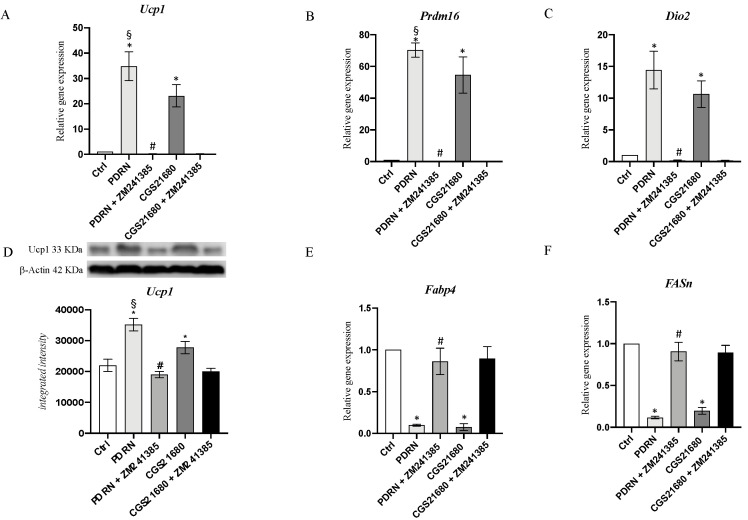
The graphs represent qPCR results of UCP1 (**A**), PRDM16 (**B**), DIO2 (**C**), FABP4 (**E**) and FASn (**F**) mRNA expression and protein levels of UCP1 (**D**) obtained from adipocytes cells. Values are expressed as the means and SD. *N* = 3 experiments; * *p* <0.0001 vs. CTRL; # *p* < 0.0001 vs. PDRN; § *p* < 0.001 vs. CGS21680.

UCP1, PRDM16 and DIO2 mRNA expression was studied to evaluate browning process stimulation following PDRN treatment. PDRN significantly increased the mRNA expression of UCP1, PRDM16 and DIO2 compared to untreated cells (*p* < 0.0001 vs. CTRL; Figure 5). Furthermore, CGS21680 stimulated the expression of genes involved in the browning process (*p* < 0.0001 vs. CTRL; Figure 5), but PDRN treatment produced a greater effect than that of CGS21680 on UCP1 and PRDM16 expression (*p* < 0.001 vs. CGS; Figure 5). The use of the A_2Ar_ antagonist ZM241385 abrogated PDRN and CGS21680 effects, further confirming A_2A_ receptor involvement in the browning process (Figure 5). Moreover, the PDRN browning effect on UCP1 protein levels was also evaluated to support mRNA expression results; in fact, the obtained data confirm that UCP1 protein expression was significantly stimulated following PDRN treatment (Figure 5D). FABP4 and FASn mRNA expression was studied as WAT markers to confirm browning process induction and the reduction of white adipocytes in favor of brown adipocytes. PDRN treatment significantly decreased both FABP4 and FASn mRNA expression compared to untreated cells (*p* < 0.0001 vs. CTRL; Figure 5). Overlapping results were obtained following CGS21680 treatment, which reduced both FABP4 and FASn mRNA expression (*p* < 0.0001 vs. CTRL; Figure 5). ZM241385 co-incubation with PDRN and CGS abrogated the effects observed following the treatment with both adenosine receptor agonists (Figure 5).

### 2.5. PDRN Modulates the Adipokine Network 

**Figure 6:**
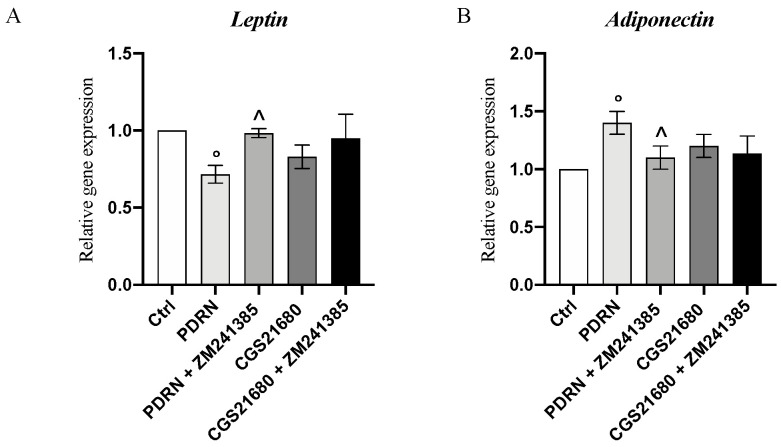
The graphs represent qPCR results of leptin (**A**)and adiponectin (**B**) mRNA expression obtained from adipocytes cells. Values are expressed as the means and SD. *N* = 3 experiments; ° *p* < 0.05 vs. CTRL; ^ *p* < 0.05 vs PDRN.

PDRN effects on adipokine mRNA expression were investigated to evaluate whether PDRN could modulate pro-inflammatory leptin and anti-inflammatory adiponectin. PDRN significantly reduced and increased leptin and adiponectin mRNA expression, respectively, compared to control cells (*p* < 0.05 vs. CTRL; Figure 6) whereas the treatment with the A_2AR_ antagonist ZM241385 abolished its effects, thus confirming the anti-inflammatory effect of PDRN through adenosine receptor modulation.

## 3. Discussion

The therapeutic approach aimed at treating obesity is mainly based on the management of insulin sensitivity, thus delaying the onset or progression of type 2 diabetes. However, recent scientific research has provided new pre-clinical evidence that highlighted the hypothesis that browning process stimulation might be exploited for the treatment of different metabolic disorders and their related diseases. In fact, brown adipocytes are involved in energy expenditure and modulate the proton gradient through uncoupling cellular respiration from mitochondrial ATP synthesis [4]. This energetic mechanism is regulated by UCP1 that regulates thermogenesis and is responsible for cAMP increase [18]. 

Adenosine is a purine nucleoside that controls several processes in different cell types and also in adipocytes due to the binding of its specific adenosine receptors. In this context, a previous study showed that adenosine may inhibit cAMP production, thus decreasing, in turn, oxygen consumption and lipolysis through A_1r_ activation [19]. On the contrary, Gnad et al. demonstrated that adenosine induces lipolysis and thermogenesis activation in brown and white adipocytes in both humans and mice, likely by A_2A_ receptor modulation [13]. In addition, recent works have shown that A_2Ar_ agonists, such as CGS21680 or PSB-0777, activate lipolysis, increase energy expenditure and oxygen consumption, improve glucose tolerance, protect from diet-induced obesity and, finally, induce browning of WAT, demonstrating adenosine A_2A_ receptor involvement in browning process induction [20,21]. In accordance with these previous studies, PDRN, a well-known A_2A_ receptor agonist, induced the browning process in white adipocytes differentiated from the 3T3L-1 cell line as demonstrated by the increase in UCP1, PRDM16 and DIO2 expression, which are all considered important targets of browning. In contrast, FASn and FABP4, two genes typically expressed in WAT, were significantly reduced following PDRN treatment and, consequently, browning induction. 

These PDRN positive browning effects were compared with CGS21680 effects, which is one of the most widely used specific A_2A_ receptor agonists, with PDRN treatment producing a greater effect than that of CGS21680 on UCP1 and PRDM16 expression. Uncoupling is a predominant feature for browning and mitochondrial biogenesis. In fact, oxygen consumption was significantly increased following PDRN or CGS21680 treatment in our experimental setting, thus demonstrating the stimulation of metabolic activity, as observed in stimulated brown adipocytes.

The browning induction was abrogated when adipocytes were also treated with the A_2A_ receptor antagonist ZM241385, thus demonstrating that the browning process was promoted through A_2A_ receptor stimulation. 

These molecular results were confirmed by the Oil Red O staining images that showed not only an increase in the trans-differentiation of white adipocytes to brown adipocytes after PDRN treatment but also a reduction in cell volume and lipid droplet size together with an increase in the number of lipid droplets and differentiation rate, which are considered features of the browning process. Adipose tissue secretes different factors and, in particular, leptin and adiponectin are produced mainly by adipocytes and are classified as adipokines [22]. Adiponectin and leptin are involved in the maintenance of glucose, lipid and energy homeostasis [23]; adiponectin is secreted exclusively from adipose tissue and is inversely associated with obesity, thus increasing adipocyte lipid storage/adipogenesis and reducing lipid accumulation [24]. Leptin plays an important role in the regulation of adipose tissue metabolism by reducing lipogenesis, enhancing triglyceride hydrolysis and by fatty acid oxidation [25]. PDRN treatment increased and reduced mRNA expression of adiponectin and leptin, respectively, compared to untreated cells. This adipokine modulation in the differentiated 3T3L-1 was abolished when cells were co-incubated with ZM241385, thus demonstrating that PDRN was not only able to modulate the adipokine network but also that this mechanism of action occurred through A_2A_ receptor stimulation. 

The results obtained to date are quite interesting and open a way to new frontiers of treatment. However, the main limitation of this study is related to the evaluation of PDRN effects in an in vitro model of browning; therefore, future in vivo studies will be needed to evaluate its efficacy. On the other hand, PDRN might be considered as a new browning agent, and since this adenosine agonist is currently on the market for different therapeutic purposes, it could be readily available for a clinical trial in obese patients. In addition, preliminary pharmacokinetics data obtained from mice demonstrated that PDRN has a half-life of approximately 12–17 h, thus suggesting that it might be suitable for daily dosing, making its administration in clinical practice easy [17]. As previously mentioned, obesity is an important risk factor for diabetes, and since PDRN is also used for the treatment of diabetic foot ulcers [26], its use might represent an appealing additional therapeutic advantage that may further justify its administration in the clinical setting of obesity. Finally, the use of centrally acting anorectic drugs, especially those boosting the serotoninergic system, has been pursued, but the occurrence of tolerance and cardiovascular side effects has limited its therapeutic use [27]. Therefore, PDRN which is well tolerated and has shown a very good safety profile in several clinical trials [28,29,30], could represent an innovative strategy to treat obese patients. 

In light of all these observations, this study has a strong translational impact. However, these preclinical results warrant investigation in an animal model of obesity and subsequently in a clinical scenario.

## 4. Materials and Methods

### 4.1. Reagents

Mouse embryo 3T3-L1 cell line was obtained from the American Type Culture Collection (ATCC, CL-173). Dulbecco’s modified Eagle medium (DMEM), fetal bovine serum (FBS), trypsin/EDTA and penicillin/streptomycin were purchased from GIBCO (BRL Life Technologies, Grand Island, NY, USA). Isobutyl-3-methylxanthine (IBMX), dexamethasone (DEX), insulin and Oil Red O staining were purchased from Sigma-Aldrich (St. Louis, MO, USA). CGS21680 and ZM241385 were obtained from Tocris Bioscience (Abingdon, UK). 

### 4.2. T3-L1 Pre-Adipocyte Cell Culture and Differentiation 

Mouse 3T3-L1 pre-adipocytes were cultured in DMEM supplemented with 4.5 g/L^−1^ glucose, 10% heat-inactivated FBS, 100 U/mL penicillin and 100 μg/mL streptomycin at 37 °C in a humidified incubator with 5% of CO_2_; the medium was replaced every 2–3 days. Upon reaching 70–90% confluence, 3T3-L1 differentiation was induced by replacing the medium with a differentiation media (MDI), composed of DMEM supplemented with 10% FBS, 0.5 mM 3-isobutyl-1-methylaxanthine, 1.0 µM dexamethasone and 1.5 µg/mL insulin. After 48 h of induction, differentiation medium was replaced with a growth medium containing DMEM supplemented with 10% FBS and 1.5 µg/mL insulin; the growth medium was replaced every 2 days until day 14. On day 14, 3T3-L1 mature adipocytes were treated with PDRN at the dose of 10 µg/mL or CGS21680 at the dose of 1 µM alone or in combination with ZM241385, an A_2A_ receptor antagonist, at the dose of 1 µM for 24 h. CGS21680 and ZM241385 doses were chosen according to a previously published paper [31].

### 4.3. Cell Viability Assay

Cell viability was measured by MTT assay. 3T3-L1 cells were seeded in 96-well plates at a density of 10 × 10^4^ cells/well and incubated at 37 °C with 5% of CO_2_ for 24 h. After incubation, cells were treated with PDRN (10 µg/mL), CGS21680 (1 µM), PDRN (10 µg/mL) + ZM241385 (1 µM) and CGS21680 (1 µM) + ZM241385 (1 µM) for an additional 24 h. Then, 5 h before the end of the treatment, 20 μL of 3-(4,5-dimethylthiazol-2yl)-2,5-diphenyl tetrazolium bromide (MTT) solution (0.5 mg/mL) was added into each well and incubated until the treatment was completed. At the end of the reaction, the MTT solution was removed, and the crystals of formazan were dissolved by adding 200 μL of DMSO per well. Cell viability was measured at 540 and 620 nm of absorbance using a VICTOR Multilabel Plate Reader (Perkin Elmer; Waltham, MA, USA). The reported data represent the percentage of cell viability compared with controls.

### 4.4. Oil Red O lipid staining

After differentiation, mature adipocytes were treated with PDRN or CGS21680 at the dose of 10 µg/mL and 1 µM, respectively, alone or in combination with ZM241385 at the dose of 1 µM for 24 h. At the end of the treatment, cells were washed twice with sterile PBS and fixed with a formaldehyde 10% for 30 min at room temperature. Fixed cells were washed with PBS to remove the excess of fixative and stained with a working Oil Red O solution (40% water and 60% Oil Red O stock solution) for 50–60 min at room temperature. After washing with distillated water, cells were observed with a Leica microscope (Milan, Italy). ImageJ was used to measure the diameters and number of lipid droplets [32]. Moreover, the differentiation rate was calculated as the number of lipid droplets containing adipocytes per total number of cells. 

### 4.5. Lipid Accumulation

After treatment with PDRN or CGS21680, the supernatant was removed from wells, cells were washed with sterile PBS and fixed with 4% formaldehyde at room temperature for 15 min. After that, cells were stained with Oil Red O (1.25 mL per well for 6-well plate) for 30 min at room temperature. Cells were then washed 5 times with distillated water and 2.5 mL per well of 100% 2-propanol was added. The plate was incubated at room temperature for 10 min in an orbital shaker. Then, 200 µL of supernatant of each well was transferred into a 96-well plate. Absorption was measured at 510 nm using a VICTOR Multilabel Plate Reader (Perkin Elmer; Waltham, MA, USA). 

### 4.6. Extracellular Oxygen Consumption Assay

To evaluate the extracellular O_2_ Consumption, cells were seeded in a 96-well plate at a density of 5 × 10^4^ cells per well and treated with PDRN and CGS21680, alone or in combination with ZM241385 for 24 h. The following day, culture medium was removed from all wells and replaced with 150 µL of fresh culture media; 10 µL of reconstituted extracellular O_2_ consumption reagent was added to each sample well, while 10 µL of fresh culture media was added to blank control wells (150 µL fresh culture media). Each well was sealed with 100 µL of pre-warmed high-sensitivity mineral oil. Extracellular oxygen consumption was measured in a fluorescent plate reader at 1.5 min intervals for 90–120 min at Ex/Em = 380/650 nm.

### 4.7. RNA Isolation, cDNA Synthesis and Real-Time Quantitative PCR Amplification

Total RNA was isolated from adipocytes for RTqPCR analysis using the Trizol Reagent Kit (Life Technologies, Monza, Italy). The first strand of cDNA was synthesized from 1 µg of total RNA using a SuperScript IV Master Mix (ThermoFisher, Waltham, MA, USA). GAPDH mRNA was used as an endogenous control to allow for the relative quantification. RTqPCR was performed to evaluate the gene expression of UCP1, PRDM16, DIO2, leptin, adiponectin, FABP4 and FASn using the BrightGreen 2X qPCR MasterMix-Rox (Applied Biological Materials, Vancouver, Canada) and the QuantStudio 6 Flex Real-Time PCR System (Applied Biosystems, CA, USA). The amplified PCR products were quantified by measuring the calculated cycle thresholds (CT) of target genes and GAPDH mRNA. After normalization, the mean value of the control group was chosen as the calibrator, and the results were expressed according to the 2^−ΔΔCt^ method, as a fold change relative to normal controls. Primers used to identify both targets and reference genes are catalogued in Table 1.

### 4.8. Western Blot Analysis

Cells were collected following 24 h of treatment and homogenized in RIPA buffer (25 mM Tris/HCl, pH 7.4; 1.0 mM EGTA; 1.0 mM EDTA) with 1% of NP40, 0.5% of phenyl methylsulfonyl fluoride (PMSF), aprotinin, leupeptin and pepstatin (10 μg/mL each). Cell suspension was centrifuged at 15,000 rpm for 15 min at 4 °C, and the supernatants were used to obtain the total protein content using the Bio-Rad protein-assay kit (Bio-Rad, Hercules, CA, USA). The remaining supernatant was mixed with Laemmli sample buffer (62 mmol/L Tris pH 6.8, 10% glycerol, 2% SDS, 5% β-mercaptoethanol and 0.003% bromophenol blue), and proteins (30 μg) were separated by electrophoresis on a 10% sodium dodecyl sulphate (SDS) polyacrylamide gel. Following separation, proteins were transferred in PVDF membranes using a specific transfer buffer (39 mmol/L glycine, 48 mmol/L Tris, pH 8.3 and 20% methanol) at 300 mA for 2 h and blocked with 5% non-fat dry milk in TBS 0.1% for 1 h at room temperature. Membranes were incubated with a primary antibody to detect Ucp1 (ab209483, 1:5000, Abcam, Cambridge, UK) diluted in TBS–0.1% Tween overnight at 4 °C. The day after, membranes were washed with TBS–0.1% Tween and incubated with a secondary peroxidase-conjugated goat anti-rabbit antibody (KPL, Gaithersburg, MD, USA) for 1 h at room temperature. After washing with TBS –0.15% Tween buffer, an enhanced chemiluminescence system (LumiGlo reserve; Seracare, Milford, MA, USA) was used to analyze membranes. The protein signal was detected and quantified by scanning densitometry using a bio-image analysis system (C-DiGit, Li-cor, Lincoln, NE, USA). Results were expressed as relative integrated intensity [33,34,35]. β-actin (Cell Signaling, Danvers, MA, USA) was used to confirm equal protein loading.

### 4.9. Statistical Analysis

The results are expressed as means ± standard deviation (SD). The reported values are the results of three experiments. All assays were performed in duplicate to ensure reproducibility. Different groups were analyzed using one-way ANOVA approach with Tukey’s post-test for intergroup comparisons. A *p* value < 0.05 was considered significant, and graphs were set using GraphPad Prism software (Version 8.0 for macOS, San Diego, CA, USA).

## 5. Conclusions

In conclusion, we demonstrated for the first time that PDRN is able to induce the white-to-brown adipose differentiation through A_2Ar_ stimulation. Since PDRN is a safe drug already available in the market for other therapeutic indications, its “anti-obesity” activity could represent an innovative strategy to treat obese patients. 

## Figures and Tables

**Table 1:** Primer list.

Gene	Forward	Reverse
GAPDH	5′ GAGTCAACGGATTTGGTCGT3′	5′ TTGATTTTGGAGGGATCTCG3′
FASn	5′ TTGCTGGCACTACAGAATGC3′	5′ AACAGCCTCAGAGCGACAAT3′
FABP4	5′ TCACCTGGAAGACAGCTCCT3′	5′ AATCCCCATTTACGCTGATG3′
Adiponectin	5′ GTTGCAAGCTCTCCTGTTCC3′	5′ TCTCCAGGAGTGCCATCTCT3′
UCP1	5′ ATGGTGAACCCGACAACTTC3′	5′ CAGCGGGAAGGTGATGATA3′
DIO2	5′ ATGGGACTCCTCAGCGTAGA3′	5′ GGAGGAAGCTGTTCCAGACA3′
PRDM16	5′ CGAGGAGGAGACCGAAGAC3′	5′ GAAGTCTGGTGGGATTGGAA3′
LEPTIN	5′ TCTTTCCGGAACATTTGGAG3′	5′ TGTGAGATCAACCCTGGACA3′

## Data Availability

The data presented in this study are available on request from the corresponding author.
